# Long‐term outcomes in patients treated with proton therapy for localized prostate cancer

**DOI:** 10.1002/cam4.1159

**Published:** 2017-09-06

**Authors:** Masaru Takagi, Yusuke Demizu, Kazuki Terashima, Osamu Fujii, Dongcun Jin, Yasue Niwa, Takashi Daimon, Masao Murakami, Nobukazu Fuwa, Tomoaki Okimoto

**Affiliations:** ^1^ Proton Therapy Center Sapporo Teishinkai Hospital Sapporo Hokkaido Japan; ^2^ Department of Radiology Hyogo Ion Beam Medical Center Tatsuno Hyogo Japan; ^3^ Department of Radiation Oncology Hakodate Goryoukaku Hospital Hakodate Hokkaido Japan; ^4^ Department of Radiology Uji‐Tokushukai Medical Center Uji Kyoto Japan; ^5^ Department of Biostatistics Hyogo College of Medicine Nishinomiya Hyogo Japan; ^6^ Center for Radiation Oncology Dokkyo Medical University Shimotsuga‐gun Tochigi Japan

**Keywords:** Adverse effects, age factors, prostate‐specific antigen, prostatic neoplasms, proton therapy, radiotherapy, recurrence

## Abstract

The aim of this retrospective study was to report long‐term clinical outcomes in patients treated with proton therapy (PT) for localized prostate cancer. Between 2001 and 2014, 1375 consecutive patients were treated with PT. Patients were classified into prognostic risk groups based on the National Comprehensive Cancer Network criteria. Freedom from biochemical relapse (FFBR), cancer‐specific survival (CSS) and incidence of late gastrointestinal (GI)/genitourinary (GU) toxicities were calculated. Multivariate analysis was performed to identify clinical prognostic factors for FFBR and late toxicities. The median follow‐up period was 70 months (range, 4–145 months). In total, 99% of patients received 74 Gy (relative biologic effectiveness [RBE]); 56% of patients received neoadjuvant androgen deprivation therapy. For the low‐, intermediate‐, high‐, and very high‐risk groups, 5‐year FFBR was 99% (95% confidence intervals [CI], 96–100%), 91% (95% CI, 88–93%), 86% (95% CI, 82–89%), and 66% (95% CI, 53–76%), respectively, and 5‐year CSS was 100% (95% CI, 100–100%), 100% (95% CI, 100–100%) , 99% (95% CI, 97–100%), and 95% (95% CI, 94–98%), respectively. Patient age, T classification, Gleason score, prostate‐specific antigen, and percentage of positive cores were significant prognostic factors for FFBR. Grade 2 or higher GI and GU toxicities were 3.9% and 2.0%. Patient age was a prognostic factor for both late GI and GU toxicities. This study represents the largest cohort of patients treated with PT for localized prostate cancer, with the longest follow‐up to date. Our results demonstrate that the biochemical control of PT is favorable particularly for high‐ and very high‐risk patients with lower late genitourinary toxicity and indicates the necessity of considering patient age in the treatment protocols.

## Introduction

Prostate cancer (PCa) is the second most common cancer in men 1. The treatment for localized PCa is selected based on a consideration of the patient age, risk group, and other factors 2. Due to prostate‐specific antigen (PSA) screening, an increasing proportion of patients are being diagnosed with localized PCa and are candidates for definitive external beam radiotherapy (EBRT) 3.

Although the prostate resides deep within the pelvis and is surrounded by several organs at risk (OARs), the technical development of X‐ray‐based radiotherapy makes it possible to deliver a high dose to the prostate while minimizing the dose to adjacent OARs. As a result, recent randomized controlled trials (RCTs) and several single‐institution studies have confirmed the advantage of high‐precision EBRT to achieve optimal biochemical control and a low rate of toxicity in patients with localized PCa 4, 5, 6, 7, 8, 9.

The physical characteristics of proton beam therapy include a Bragg peak and reduced lateral scatter, which enable more conformal dose distribution compared with that of X‐ray‐based radiotherapy. The effectiveness of proton therapy (PT) for PCa has been investigated for more than 10 years, but whether the basic difference in radiation dose distribution between X‐rays and proton beams can be involved in the improvement of clinical outcomes has not been established. The results of RCTs directly comparing PT with modern X‐ray‐based radiotherapy have not been reported, and nonrandomized studies have reported mixed results.

In this study, we analyzed the long‐term outcomes of PT for localized PCa compared with those of other EBRTs.

## Materials and Methods

### Study design and patients

We conducted an Institutional Review Board‐approved, retrospective analysis of patients with localized PCa who received definitive PT between April 2001 and May 2014 at Hyogo Ion Beam Medical Center. The five inclusion criteria for this study were as follows: (1) histologically confirmed prostate cancer; (2) Eastern Cooperative Oncology Group performance status ≤2; (3) adequate organ function; (4) no castration‐resistant prostate cancer; and (5) duration of follow‐up ≥24 months for survivors. A total of 1375 patients were enrolled. All eligible patients provided written informed consent before treatment.

The pretreatment workup included medical history, PSA testing, computed tomography (CT) scans from abdomen to pelvis, magnetic resonance imaging (MRI) of prostate, bone scintigraphy, and in‐house pathology review of prostate biopsy specimens to verify the Gleason score (GS).

Patients were classified into four risk groups as defined by the National Comprehensive Cancer Network (NCCN) criteria according to T classification, GS, and the PSA level at diagnosis excepting the very low‐risk group 10. Patient and treatment characteristics are shown in Table 1.

**Table 1:** Characteristics of patients and treatments

Characteristics	No.	%
Total	1375	
Age, median year [range]	69 (44–92)	
ECOG PS 0/1/2	1223/143/9	89/10/1
T classification 1/2/3/4	513/643/213/6	37/47/15/<1
Gleason Score ≤ 6/7/≥ 8	426/668/281	21/49/20
Initial PSA, median [range]	9.1 [0.6–341.0]	
<10.0/10.0‐20.0/>20.0	769/342/264	56/25/19
Percent Core positivity, median [range]	30 [2–100]	
0‐24/25‐49/50‐74/75‐100	516/451/273/135	37/33/20/10
NCCN risk groups		
Low/Intermediate/High/Very high	249/602/449/75	18/44/33/5
Total dose		
74.0 Gy (RBE)/78.0 Gy (RBE)	1363/12	99/1
Neoadjuvant ADT no/yes	595/780	43/57
Period, median month [range]	7 [1–84]	
Low/Intermediate/High/Very high	63/264/380/73	25/44/85/97
Period, median month	7/7/7/7	
Concurrent ADT no/yes	1279/96	93/7
Period, median month [range]	2 [1–3]	
Low/Intermediate/High/Very high	1/9/62/24	<1/1/14/32
Period, median month	2/2/2/2	
Adjuvant ADT no/yes	1316/59	96/4
Period, median month [range]	20 [2–96]	
Low/Intermediate/High/Very high	0/2/42/16	0/<1/9/21
Period, median month	0/34/20/19	
Total ADT no/yes	590/785	43/57
Period, median month [range]	7 [1–128]	
Low/Intermediate/High/Very high	64/265/382/74	26/44/85/99
Period, median month	7/7/7/9	
Anticoagulant drugs no/yes	1233/142	90/10
Diabetes mellitus no/yes	1230/145	89/11

ECOG PS, Eastern Cooperative Oncology Group performance status; PSA, prostate‐specific antigen; NCCN, National Comprehensive Cancer Network; Gy (RBE), grays relative biological effectiveness; ADT, androgen deprivation therapy.

### Proton therapy treatment

Fused CT and MRI images were used to define both the clinical target volume (CTV) and OARs. The CTV included the whole prostate. The base of the seminal vesicles was included in the CTV for the patients who satisfied at least one of the following criteria: (1) T3a or higher; (2) GS ≥ 8; and (3) PSA ≥ 20 ng/mL. For the patients with T3b or PSA ≥ 50 ng/mL, the entire seminal vesicle structure was included in the CTV. The planning target volume (PTV) consisted of the CTV and a 10‐mm margin in all directions, except posteriorly, where the margin was reduced to 7 mm.

Proton beams were produced using passive scatter methods. Beam arrangements of PT were performed using bilateral beams, and single beam PT treatment given once daily, alternating sides for each day. A relative biological effectiveness (RBE) value for PT of 1.1 was applied. The doses of PT are reported as Gy (RBE), which is defined as the physical doses multiplied by the RBE 11. All patients were treated with 2 Gy (RBE) fraction per day. The dose constraints were as follows: (1) minimum dose of CTV ≥ 70 Gy (RBE); (2) the volume of the CTV + 5 mm that received 95% of the prescribed dose was ≥90%; (3) the 3‐mm internal wall volume of the rectum that received 65 Gy (RBE) (V65) was ≤17% and 40 Gy (RBE) (V40) was ≤35%; (4) the V65 and V40 of the 3‐mm internal bladder wall were ≤25% and ≤50%; and (5) the maximum dose of the large bowel was ≤61 Gy (RBE) and small bowel was ≤55 Gy (RBE). Portal images were used to verify the position by matching to bony structures. Fiducial markers inside prostate and rectal balloons were not used.

### Androgen deprivation therapy

Neoadjuvant androgen deprivation therapy (N‐ADT) for a duration of 6 months was required if the patient exhibited at least one of the following four conditions: (1) T2c or higher; (2) GS ≥ 8; (3) PSA ≥ 20 ng/mL; and 4) percentage of positive cores >50%. Patients with T4 or PSA 50 ≥ ng/mL received concurrent ADT. There were no protocol regulations for adjuvant ADT.

### Follow‐up evaluation

The follow‐up evaluations were performed at intervals of 3 months for 5 years and 6 months thereafter. Every follow‐up included PSA testing and evaluation of late toxicities. Biochemical relapse was analyzed using the Phoenix definition 12. Clinical recurrence was based on available clinical, histological, or radiographic evidence of disease recurrence or metastases. Late gastrointestinal (GI) and genitourinary (GU) toxicities were graded according to the National Cancer Institute Common Terminology Criteria for Adverse Events, version 4.0 13.

### Statistical analyses

Continuous and categorical data are summarized as medians with ranges (minimums–maximums) and as frequencies with percentages, respectively. The rate of freedom from biochemical relapse (FFBR), cancer‐specific survival (CSS), overall survival (OS) and incidence of late GI/GU toxicities were estimated using the Kaplan–Meier method and compared with the log‐rank test. All endpoints were calculated from the PT completion date. The Cox proportional hazards model was used for multivariable analysis of possible associations between FFBR and late GI/GU toxicities with prognostic factors. The following factors were tested for FFBR: patient age, T classification, percentage of positive cores, GS, PSA, and use of ADT with a total duration of more than 6 months. Patient age, use of ADT with a total duration of more than 6 months, use of anticoagulant drugs, and diabetes mellitus were tested with grade 2 or higher late GI/GU toxicities. A *P* ≤ 0.05 was considered statistically significant. These statistical analyses were performed using SPSS Statistics 22 software (IBM, Armonk, NY). The 95% confidence intervals (95% CIs) for the FFBR, OS rate, and incidence of late GI/GU toxicities were calculated using EZR 14.

## Results

### Patients

The median follow‐up period was 70 months (range, 4–145 months). According to NCCN risk groups, 249 (18%), 602 (44%), 449 (33%), and 75 (5.5%) of patients were classified as low‐, intermediate‐, high‐, and very high‐risk groups, respectively. The prescribed dose was 74 Gy (RBE) in 1363 patients (99%) and 78 Gy (RBE) in 12 patients (<1%). A total of 780 patients (56%) were treated with N‐ADT with a median duration of 7 months (range, 1–84 months). Concurrent ADT was given to a total of 96 patients (7.0%) with a median duration of 2 months (range, 1–3 months). Adjuvant ADT was given to a total of 59 patients (4.3%) with a median duration of 20 months (range, 2–96 months).

### Disease control

Biochemical relapse occurred in 177 patients (13%). The median time to biochemical relapse was 39 months (range, 2–119 months). According to NCCN risk groups, the median times to biochemical relapse were 65 months (range, 21–87 months) in low‐risk groups, 48 months (range, 6–114 months) in intermediate‐risk, 32 months (range, 2–119 months), in high‐risk, and 33 months (range, 9–112 months) in very high‐risk. Of 177 patients who experienced biochemical relapse, 49 patients (28%) experienced biochemical relapse more than 5 years after PT.

**Figure 1:**
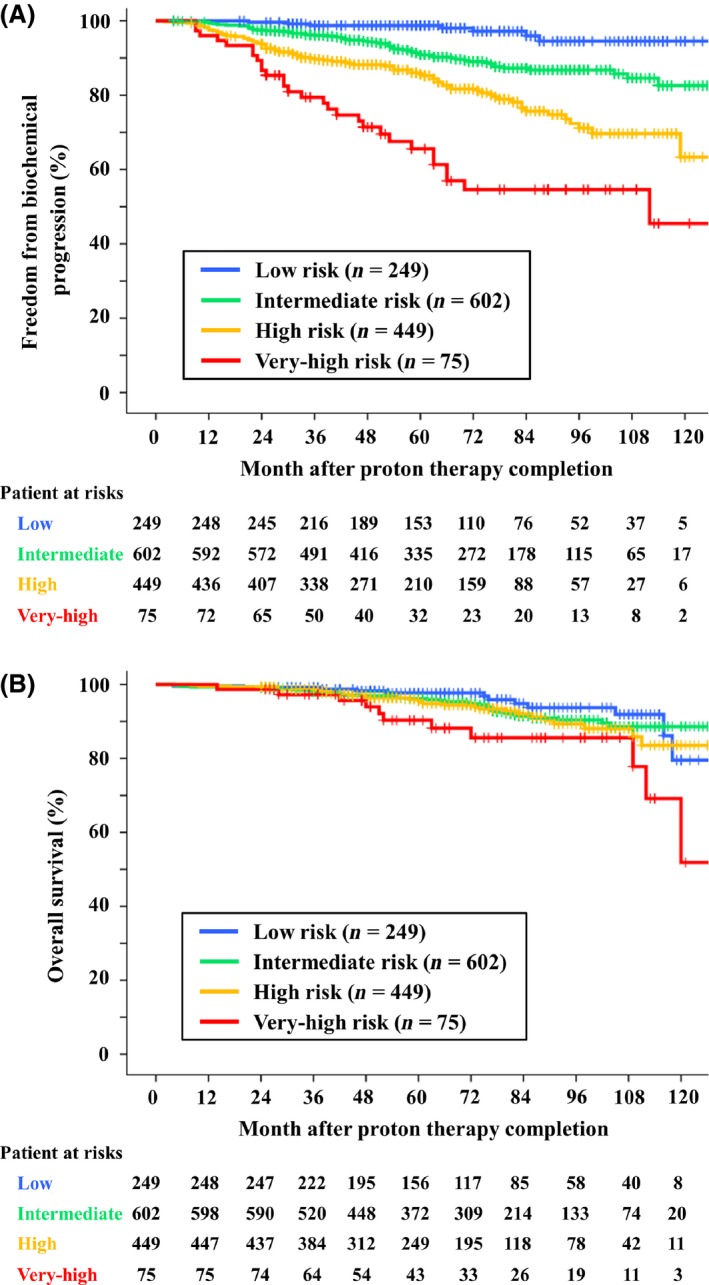
Freedom from biochemical relapse (A) and overall survival (B) are shown according to National Comprehensive Cancer Network risk groups.

The 5‐ and 8‐year FFBR rates for all patients were 89% (95% CI, 87–91%) and 82% (95% CI, 79–84%). According to NCCN risk groups, the 5‐ and 8‐year FFBR rates were 99% (95% CI, 96–100%) and 95% (95% CI, 88–98%) for low‐risk patients, 91% (95% CI, 88–93%) and 87% (95% CI, 83–90%) for intermediate‐risk, 86% (95% CI, 82–89%) and 71% (95% CI, 64–77%) for high‐risk, and 66% (95% CI, 53–76%) and 55% (95% CI, 41–67%) for very high‐risk, respectively (Fig. 1A). The FFBR rate for very high‐risk patients was significantly lower than those of low‐, intermediate‐, and high‐risk groups (*P* < 0.001, *P* < 0.001, and *P* < 0.001). The mean 5‐ and 8‐year FFBR rates for high‐ and very high‐risk patients were 83% (95% CI, 78–87%) and 68% (95% CI, 61–76%).

Multivariate analysis identified T classification, GS, initial PSA, and percentage of positive cores as significant prognostic factors for FFBR (Table 2). Patient age was a strong prognostic factor for FFBR. Contrary to expectations, younger patients tended to be more likely to experience biochemical relapse than older patients. The rate of biochemical relapse for patents ≤64 years of age was more than twice as high as that of patients ≥70 years of age.

**Table 2:** Multivariate analyses of freedom from biochemical relapse

Variable		*n*	HR	95% CI	*P*
Patient age	≤64	418	1.000	(ref)	—
	65–69	352	0.687	0.474–0.996	0.047
	70–74	359	0.427	0.279–0.653	<0.001
	≥75	246	0.465	0.295–0.733	0.001
T classification	T1	820	1.000	(ref)	—
	T2	341	1.696	1.138–2.528	0.009
	T3‐4	214	1.911	1.191–3.064	0.007
Percentage of positive cores (%)	0–24	516	1.000	(ref)	—
	25–49	451	1.905	1.208–3.003	0.006
	50–74	273	2.298	1.390–3.797	0.001
	75–100	135	2.854	1.642–4.961	<0.001
Gleason Score	≤ 6	426	1.000	(ref)	–
	7	668	0.999	0.653–1.529	0.997
	≥8	281	1.739	1.075–2.815	0.024
PSA (ng/ml)	<10.0	769	1.000	(ref)	–
	10.0–20.0	342	2.114	1.432–3.120	<0.001
	>20.0	264	2.408	1.531–3.789	<0.001
Total ADT (month)	≥6	699	0.697	0.474–1.025	0.067

PSA, prostate‐specific antigen; ADT, androgen deprivation therapy.

In total, 43 patients (3.1%) experienced clinical recurrence, including 11 local recurrences, 15 pelvic lymph node metastases, 18 bone metastases, and 3 others.

### Survival

At the last individual follow‐up, 91 patients (6.6%) had died; 12 patients had died from prostate cancer, 76 from coincident diseases, and 3 from unknown causes.

The 5‐ and 8‐year CSS rates were 100% (95% CI, 100–100%) and 100% (95% CI, 100–100%) for low‐risk patients, 100% (95% CI, 100–100%) and 99% (95% CI, 97–100%) for intermediate‐risk, 99% (95% CI, 97% to 100%) and 98% (95% CI, 95% to 99%) for high‐risk, and 95% (95% CI, 94% to 98%) and 92% (95% CI, 81% to 97%) for very‐high‐risk, respectively. The CSS rate for very high‐risk patients was significantly worse than those of low‐, intermediate‐ and high‐risk groups (*P* < 0.001, *P* < 0.001, and *P* = 0.014).

The 5‐ and 8‐year OS rates were 98% (95% CI, 93–99%) and 94% (95% CI, 88–97%) for low‐risk patients, 96% (95% CI, 94–98%) and 90% (95% CI, 87–93%) for intermediate‐risk, 96% (95% CI, 93–97%) and 89% (95% CI, 84–93%) for high‐risk, and 90% (95% CI, 80–96%) and 86% (95% CI, 73–93%) for very‐high‐risk, respectively (Fig. 1B). The OS rate for very high‐risk patients was significantly worse than those of low‐, intermediate‐, and high‐risk groups (*P* = 0.003, *P* = 0.010, and *P* = 0.047).

### Late toxicities

**Figure 2:**
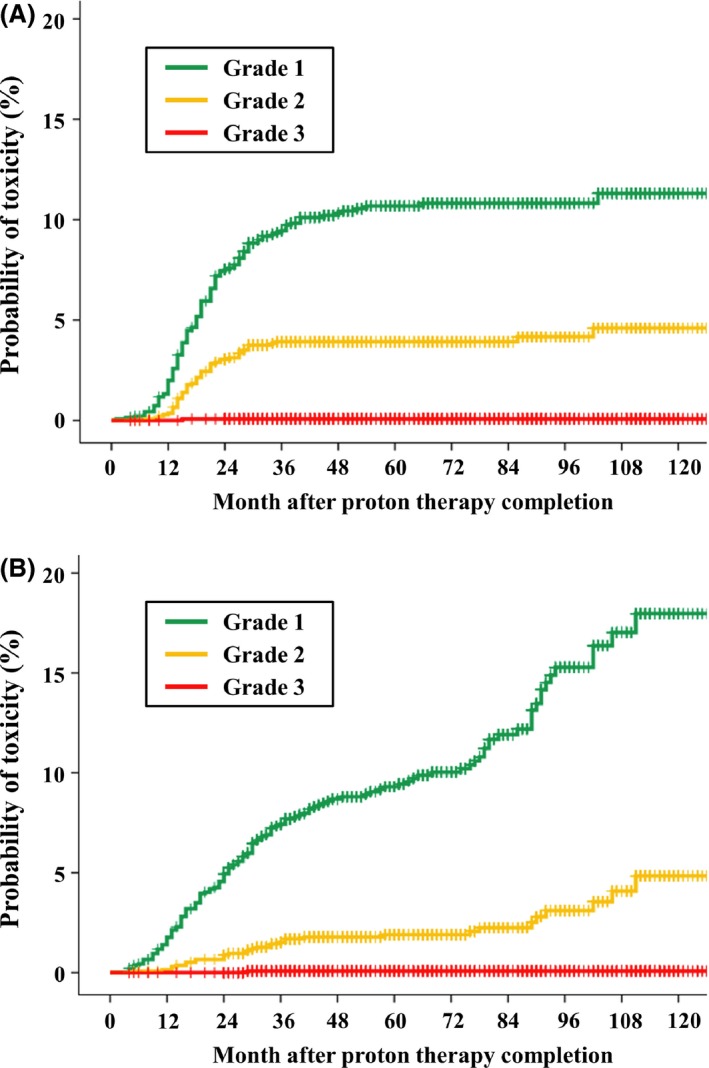
Incidence of late gastrointestinal toxicity (A) and late genitourinary toxicity (B).

With respect to late GI toxicity, grade 1 events were observed in 82 patients and grade 2 events in 53 patients. One patient experienced grade 3 rectal bleeding, which required blood transfusion and hyperbaric oxygen therapy. The grade 1, grade 2, and grade 3 late GI toxicity rates at 5 years were 10% (95% CI, 8.5–12%), 3.8% (95% CI, 2.8–4.8%), and 0.1% (95% CI, 0–0.2%), respectively (Fig. 2A). Multivariate analysis identified patient age and diabetes mellitus as prognostic factors for grade 2 or higher late GI toxicity (Table 3A).

**Table 3:** (A) Multivariate analyses of grade 2 or higher late gastrointestinal toxicity. (B) Multivariate analyses of grade 2 or higher late genitourinary toxicity

Variable		*n*	HR	95% CI	*P*
(A)					
Patient age	≤64	418	1.000	(ref)	—
	65–69	352	2.209	0.886–5.507	0.089
	70–74	359	2.996	1.252–7.168	0.014
	≥75	246	3.147	1.241–7.981	0.016
Total ADT (month)	≥6	699	0.941	0.544–1.626	0.826
Anticoagulant drugs	Yes	142	1.676	0.839–3.347	0.143
Diabetes mellitus	Yes	145	2.289	1.203–4.355	0.012
(B)					
Patient age	≤64	418	1.000	(ref)	—
	65–69	352	1.224	0.325–4.608	0.766
	70–74	359	1.698	0.491–5.872	0.403
	≥75	246	5.055	1.626–15.714	0.005
Total ADT (month)	≥6	699	1.466	0.678–3.168	0.331
Anticoagulant drugs	Yes	142	1.942	0.833–4.529	0.125
Diabetes mellitus	Yes	145	1.707	0.700–4.162	0.240

ADT, androgen deprivation therapy.

Grade 1 late GU toxicity events were observed in 119 patients and grade 2 events were observed in 33 patients. One patient experienced a grade 3 urethral stricture that required urinary diversion surgery. The grade 1, grade 2, and grade 3 late GU toxicity rates at 5 years were 8.9% (95% CI, 7.3–10%), 1.9% (95% CI, 1.1–2.6%), and 0.1% (95% CI, 0.1–0.2%), respectively (Fig. 2B). Only patient age was a prognostic factor for grade 2 or higher late GU toxicity (Table 3B).

The incidence of late GU toxicities continued to increase beyond 5 years, whereas the incidence of late GI toxicities had plateaued by 5 years.

## Discussion

This study represents the largest cohort of patients treated with PT for localized PCa, with the longest follow‐up to date, although these differences are small compared to previous studies. There are three notable findings. First, the results of biochemical control were favorable, particularly for high‐ and very high‐risk patients. Second, the incidences of late toxicities were very low, particularly in late GU toxicity. Third, patient age influenced both biochemical control and late GI/GU toxicities.

### Biochemical control comparison with other external beam radiotherapies

This study reports 5‐year FFBR rates of 99%, 91%, 86%, and 66% for low‐, intermediate‐, high‐, and very high‐risk patients, respectively. Table 4 shows the treatment results of the other studies using PT and EBRTs 4, 5, 6, 7, 8, 9, 15, 16, 17, 18, 19, 20. Mendenhall et al. reported that the 5‐year rates of biochemical and clinical freedom from disease progression were 99%, 99%, and 76% for low‐, intermediate‐ and high‐risk patients in three prospective trials using PT 19. Bryant et al. recently reported the results for 1327 patients treated with ≥78 Gy (RBE) using PT 20. The 5‐year biochemical control rates were 99%, 94%, and 74% for low‐, intermediate‐, and high‐risk patients, respectively. The biochemical control rates observed in our study are similar to those of other PT studies.

**Table 4:** Comparison of our findings with those of other studies

Reference	Technique	n	Median F/U (M)	Dose (Gy or Gy [RBE])	ADT rate (%)	Biochemical control (%)				Late complications (%)		
						Year	L	I	H+VH	Year	≥G2 GI	≥G2 GU
Dearnaley 2007 4	3D‐CRT	421	63	64	100	5	79	70	43	5	24	8.0
		422	64	74	100	5	85	79	57	5	33	11
Kuban 2008 5	3D‐CRT	151	104	70	0	8	63	76	26	10	13	8.0
		150	104	78	0	8	88	86	63	10	26	13
Martin 2009 15	3D‐CRT	259	68	79.8	14/11/46	5	88	77	78	NR	4.3	8.6
Zelefsky 2006 6	IMRT	561	84	81	34/52/92	8	85	76	72	8	1.7	12
Kupelian 2007 7	IMRT	770	45	70	60	5	94	83	72	5	7.0	7.0
Cahlon 2008 8	IMRT	478	53	86.4	66	5	98	85	70	5	3.4	16
Spratt 2013 9	IMRT	1001	66	86.4	28/48/91	7	99	86	68	7	4.4	21
Zietman 2010 16	X + Proton	197	66	70.2	0	5	60	63	—	NR	8.0	18
		195	66	79.2	0	5	81	80	—	NR	17	20
Johansson 2012 17	X + Proton	278	57	70	22/45/76	5	100	95	74	5	10	9.0
Ishikawa 2012 18	Carbon	927	43	57.6–66	0/100/100	5	90	97	88	5	1.9	6.3
Mendenhall 2014 19	Proton	211	62	78–82	11/9/100	5	99	99	76	5	(1.0)^1^	(0.5)^1^
Bryant 2016 20	Proton	1327	66	78	7/10/66	5	99	94	74	5	(0.6)^1^	(2.9)^1^
Present study 2016	Proton	1375	70	74–78	25/44/87	5	99	91	83	5	3.9	2.0

3D‐CRT, three‐dimensional conformal radiotherapy; IMRT, intensity‐modulated radiation therapy; X + Proton, combination of X‐ray‐based radiotherapy and proton therapy; Carbon, carbon ion therapy; Proton, proton therapy; F/U, follow‐up; Fr, fractionations; ADT, androgen deprivation therapy; L, low‐risk group; I, intermediate‐risk group; H+VH, high‐risk group, and very high‐risk group; ≥G2 GI, grade 2 or higher late gastrointestinal toxicities; ≥ G2 GU, grade 2 or higher late genitourinary toxicities; NR, not reported. ^1^Grade 3 or higher late toxicities.

For intensity‐modulated radiotherapy (IMRT), Spratt et al. reported results for 1002 patients with localized PCa treated with 86.4 Gy 9. The 7‐year biochemical control rates were 98.8%, 85.6%, and 67.9% for low‐, intermediate‐, and high‐risk patients, respectively.

In this study, the prescribed dose was not particularly high and the rate of combined use of adjuvant ADT was low. However, favorable FFBR rates were observed particularly for high‐ and very high‐risk patients, compared to those of three‐dimensional conformal radiotherapy and IMRT. Several factors may contribute to these results. In PT, although the RBE value of 1.1 is applied to all tumors, more favorable local controls than expected were observed for radiation‐resistant tumors 21, 22, 23. The estimated RBE value for prostate cancer could be higher than 1.1. Other biological studies have indicated that the RBE value is higher near the distal end of the spread‐out Bragg peak than at the center and proximal parts of the spread‐out Bragg peak 24, 25. As a PCa tends to develop in the peripheral zone of the prostate, PT using bilateral beams might consequently produce a mild‐dose escalation in some patients.

According to the results of this study, the FFBR for low‐ and intermediate‐risk patients was both over 90%. PT using 74 Gy (RBE) is an appropriate dose for low‐ and intermediate‐risk patients. However, there still seems to be room for improvement in the FFBR of high‐ and very high‐risk patients. As the incidence of late GI and GU toxicities was relatively low compared with those found in previous studies, dose escalation for high‐ and very high‐risk patients is considered feasible. Additionally, as there were few cases of the combined use of adjuvant ADT in this study, we believe that the adequate use of adjuvant ADT could improve the FFBR for high‐ and very high‐risk patients.

In this study, very high‐risk group showed significantly poorer results compared to high‐risk group, as the statistically significant differences were shown not only in FFBR but also CSS and OS. To date, the results of all patients with risk factors that were worse than intermediate‐risk have mainly been reported under the “high risk” group. However, as the “high risk” group consists of patients with varying levels of poor risk factors, it is noted that this grouping makes it difficult to determine a unified treatment policy. We propose that the treatment results of very high‐risk groups should be reported separately from those of high‐risk groups.

Although the median follow‐up of 70 months in this study was slightly longer than that in previous studies, prostate cancer is a slow‐growing tumor with the ability to recur over a prolonged period. In this study, 28% patients experienced biochemical relapse more than 5 years after PT. In low‐ and intermediate‐risk groups, the median times to biochemical relapse were longer compared to high‐ and very high‐risk groups. Therefore, longer follow‐up will be necessary to confirm these results, especially for patients in low‐ and intermediate‐risk groups.

### Late toxicity compared with other external beam radiotherapies

In this study, 5‐year grade 2 or higher late GI and GU toxicity rates were 3.9% and 2.0%, respectively. In other PT studies, Mendenhall et al. and Bryant et al. noted very low incidences of grade 3 late GI and GU toxicities 19, 20. The reported incidences of late GI toxicity of PT are comparable to those of IMRT, but late GU toxicities of PT appear to be lower than those of IMRT.

Several planning studies comparing PT and IMRT have demonstrated benefits in reducing dosage of the surrounding OARs of PT over IMRT within the low‐ to medium‐dose range of radiation rather than the high‐dose range 26, 27. Dose‐volume histogram studies have revealed that a higher radiation dose to the rectum is predominantly associated with the late GI toxicity. Conversely, a wider range of doses to the bladder is relevant to late GU toxicity 28, 29. These findings could explain not only the similar incidence of GI toxicity in PT and IMRT but also the lower incidence of GU toxicity of PT compared to IMRT.

No prospective trials comparing toxicities between PT and IMRT have been published, but a few retrospective comparison studies have reported no significant differences in late GI and GU toxicities between PT and IMRT within 2 years 30, 31. However, we observed that the incidence of GU toxicity continued to increase even beyond 5 years. This result indicates that 2 years or less is insufficient to compare the incidence of GU toxicity.

In this study, the fiducial markers in the prostate were not utilized, and only bony structures were employed to verify the position. In the modern techniques of X‐ray‐based radiotherapy, image guidance using fiducial markers or cone beam CT has become the standard method 32. The combined use of these image guidance techniques with PT may provide more precise radiotherapy, making it possible to reduce the dose of OARs and increase the dose to the target. Additionally, although only one field was irradiated on each day in this study, there are some possibilities that irradiating both fields a day might reduce the late toxicities.

Although the reported treatment results of PT including this study are favorable, RCTs directly comparing the efficacy and toxicities of PT and other EBRTs are warranted. Several RTCs comparing PT and IMRT are currently underway.

### Influence of patient age

Prognostic factor analysis revealed that younger age was a strong prognostic factor favoring biochemical relapse. As published studies on the relationship between age and biochemical control are limited, it remains unclear if this result is specific to PT or not. Only one published IMRT study noted a higher biochemical relapse rate among patients less than 65 years 9. One hypothesis of the relationship is that PCa with higher androgen density in younger patients leads to radiation resistance compared to older patients. However, in this study, the androgen density was not measured, and this result warrants further study.

Prognostic factor analysis demonstrated that the incidences of grade 2 or higher late GI and GU toxicities were higher in older patients. Budaeus et al. reviewed late toxicities following radiation therapy and reported that older age was a significant prognostic factor for late GI toxicity 33. Ahmed et al. reported that age >68 years was associated with late grade 2 GU toxicity and advocated risk‐adapted dose constraints based on patient age 34.

A recent risk‐modeling comparison study estimated a 26–39% risk reduction in a second malignancy for PT over IMRT 35. As the spot scanning method can reduce neutron scatter compared with passive scattering, PT using spot scanning may represent a favorable treatment for younger patients. However, as shown in the prognostic factor analysis, as younger patients tend to experience more biochemical relapse and less late toxicity, personalized treatment based on patient age is recommended.

### Study strengths and limitations

The strengths of this study are the large cohort size, long follow‐up period, and consistent patient treatment and evaluation at a single institution. However, this retrospective study also has several limitations. Firstly, this study did not evaluate late sexual toxicities. Second, the relationships between late toxicities and several reported risk factors such as international prostate symptom score and urinary intervention were not shown. Third, adjuvant ADT regulations were not available. Forth, androgen density was not measured.

## Conclusions

The long‐term treatment results of this study indicate that the biochemical control of PT is favorable for high‐ and very high‐risk patients. The incidence of late GU toxicity is significantly lower compared with other EBRTs. Our findings indicate the necessity for considering patient age in the treatment protocol.

## Conflicts of Interest

None declared.
